# Psychiatric morbidity and poor follow-up underlie suboptimal functional and survival outcomes in Huntington’s disease

**DOI:** 10.1186/s12883-020-01671-x

**Published:** 2020-03-12

**Authors:** Nikhil Ratna, Nitish L. Kamble, Sowmya D. Venkatesh, Meera Purushottam, Pramod K. Pal, Sanjeev Jain

**Affiliations:** 1 Department of Clinical Neuro Sciences, National Institute of Mental Health and Neuro Sciences (NIMHANS), Bangalore, India; 2 Department of Neurology, National Institute of Mental Health and Neuro Sciences (NIMHANS), Bangalore, India; 3 Molecular Genetics Laboratory, Department of Psychiatry, National Institute of Mental Health and Neuro Sciences (NIMHANS), Bangalore, India

**Keywords:** Huntington’s disease, CAG repeat length, Outcomes, Total functional capacity, Psychiatric morbidity, Suicidal tendency

## Abstract

**Background:**

Huntington’s disease (HD), an inherited, often late-onset, neurodegenerative disorder, is considered to be a rare, orphan disease. Research into its genetic correlates and services for those affected are inadequate in most low-middle income countries, including India. The apparent ‘incurability’ often deters symptomatic and rehabilitative care, resulting in poor quality of life and sub-optimal outcomes. There are no studies assessing disease burden and outcomes from India.

**Methods:**

We attempted to evaluate individuals diagnosed to have HD at our tertiary-care center between 2013 and 2016 for clinical symptoms, functionality, mortality, follow up status through a structured interview, clinical data from medical records and UHDRS-TFC scoring.

**Results:**

Of the 144 patients, 25% were untraceable, and another 17 (11.8%) had already died. Mean age at death and duration of illness at the time of death, were 53 years and 7 years respectively, perhaps due to suicides and other comorbidities at an early age. The patients who could be contacted (*n* = 81) were assessed for morbidity and total functional capacity (TFC). Mean CAG repeat length and TFC score were 44.2 and 7.5 respectively. Most individuals (66%) were in TFC stage I and II and could perhaps benefit from several interventions. The TFC score correlated inversely with duration of illness (*p* < 0.0001). The majority were being taken care of at home, irrespective of the physical and mental disability. There was a high prevalence of psychiatric morbidity (91%) including suicidal tendency (22%). Three of the 17 who died had committed suicide, and several other families reported suicidal history in other family members. Only about half the patients (57%) maintained a regular clinical follow-up.

**Conclusions:**

This study demonstrates the poor follow-up rates, significant suicidality and other psychiatric symptoms, sub-optimal survival durations and functional outcomes highlighting the need for holistic care for the majority who appear to be amenable to interventions.

## Background

Huntington’s disease (HD) is a heritable, neurodegenerative disorder, caused by an unstable trinucleotide (CAG) repeat expansion in exon 1 of the huntingtin gene on the short arm of chromosome 4 [1]. It is characterized by the classical triad of motor abnormalities, behavior problems and cognitive dysfunction [2, 3]. The diagnosis is confirmed by genetic testing, and though CAG repeat length has a strong correlation with age at onset, rate of progression and overall outcomes, it accounts for only 60–70% of the variability [4]. Several other genetic and environmental factors (including access to medical care) may be relevant as disease modifiers [5]. In spite of the availability of HD diagnostics, definitive treatment that can reliably halt or reverse the disease progression eludes us.

Historically, HD has been predominantly reported from, and studied, in populations of European and American origin [6], but now there are multiple reports from the rest of the world including the Asian region [7]. In India, HD is still under-recognized and under-reported. Though several reports have been published [8–13], the exact prevalence is difficult to estimate. An increase in the number of HD cases is being noted lately, with wider availability of genetic diagnostics. It is quite likely that the prevalence rate will be similar to that in European populations with similar haplotype [14] (approximately 3–5/1,00,000 population; or about 40,000–70,000 individuals with HD in India).

Family members most often provide care for HD patients in India; with the welfare and specialist services for patients and their families being almost non-existent (as in most low-middle income countries). Indeed, the outcomes of those diagnosed with HD have not been systematically evaluated. Thus, both cross-sectional and longitudinal studies of HD from India are needed for a holistic understanding of the disease phenotype, progression, outcomes and the factors affecting them; to enable better delivery of care. In this report, we present the disease profile and outcomes of HD patients under follow-up at a tertiary care center in India.

## Methods

Patients diagnosed to have HD by genetic analyses between 2013 and 2016 at the Genetic Counseling and Testing Clinic (GCAT), National Institute of Mental Health and Neuro Sciences (NIMHANS), were contacted over a period of 3 months (June to August, 2018), either telephonically or during follow-up visits, for a semi-structured interview (supplementary file 1). This clinical audit was part of a doctoral dissertation on Huntington’s disease which has been approved by the Institute Ethics Committee. An informed consent was obtained from all the participants willing to be interviewed.

Demographic data and clinical details were obtained from the hospital medical records. The Unified Huntington Disease Rating Scale (UHDRS) [15] total functional capacity (TFC) score [16] was used for cross-sectional assessment of the patients’ functional status. The cumulative symptom burden of patients during the course of the disease was estimated using a semi-structured questionnaire that included all the parameters of UHDRS behavioral section (for categorical scoring), prevalence of comorbidities, gross motor abilities. The current treatment status, and follow-up details were also recorded.

Summary statistics are presented as frequencies and proportions for categorical variables, and as mean and standard deviations for continuous variables. Associations between quantitative variables were studied using Pearson correlation analysis. A ‘p’ value < 0.05 was considered to be statistically significant.

## Results

**Fig. 1:**
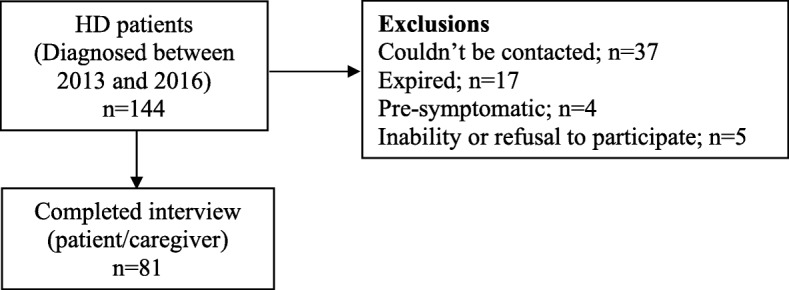
Study flow diagram

A total of 144 patients tested positive for HD at our center between 2013 and 2016. A significant proportion (37; 25%) of them could not be contacted for the present study. The reasons included inadequate contact details in hospital records, failure to come for follow-up visits, and no response in-spite of multiple attempts to contact them. Of the other 107 patients, 17 had died, 4 were still in pre-symptomatic stage (UHDRS DCL-0 in their last visit and current health status was confirmed over telephonic interview), and 5 patients were unable to or refused to participate in the study. Thus, 81 patients (and/or caregivers) were administered the full questionnaire; and caregivers of the 17 patients who had died were interviewed for the mortality profile (Fig. 1).

The mean age at symptom onset was 40.3 ± 10.4 (range 14–64) years and the mean duration of illness was 7.8 ± 4.0 (range 2–20) years; both males and females were equally represented (females: males = 0.96: 1). Geographically, 47 (58%) of these patients were from southern India (the states of Karnataka, Kerala, Tamil Nadu, Telangana and Andhra Pradesh), 15 (18%) were from West Bengal, and others from the rest of India. The informant for the interview, as a care giver, was most often a first-degree relative (61; 75%) or a second-degree relative (11; 14%); while in 9 (11%) cases, the questionnaire was answered exclusively by the patient. The patient characteristics are summarized in Table 1.

**Table 1:** Characteristics of study population (*n* = 81)

Characteristic	Observed value
Gender (females: males)	0.96: 1
Age at symptom onset (years, mean ± SD, range)	40.3 ± 10.4 (14–64)
Duration of illness (years, mean ± SD, range)	7.8 ± 4.0 (2–20)
CAG repeat length (mean ± SD, range)	44.2 ± 4.5 (39–63)
UHDRS TFC score (mean ± SD, range)	7.5 ± 3.8 (0–13)

The mean CAG repeat length was 44.2 ± 4.5 (range 39–63) repeats. Age at onset had a significant inverse correlation with CAG repeat length (correlation coefficient − 0.607, *p* < 0.0001). Fifty (61.7%) patients had motor symptoms at the onset, 25 (30.8%) had non-motor symptoms (behavioural and cognitive), while the rest (6, 7.4%) had mixed symptoms. The cumulative symptom profile of the study population is presented in Table 2. Seventy-four (91.4%) patients reported at least one behavioural/psychiatric problem warranting pharmacotherapy.

**Table 2:** Clinical profile of study population (*n* = 81)

Clinical feature	N (%)
*Motor symptoms*	
Mobility	
Walking without support	68 (84%)
Walking with support	6 (7.4%)
Bedridden	7 (8.6%)
Falls (among those who can walk)	
No falls	46 (56.8%)
Infrequent falls (< 3/week)	15 (18.5%)
Frequent falls (≥3/week)	13 (16%)
Dysarthria	56 (69%)
Dysphagia	28 (34.6%)
*Behavioral/Psychiatric symptom*	
Anger outbursts	53 (65.4%)
Low mood/Depressive symptoms	43 (53%)
Generalized anxiety	39 (48.1%)
Apathy	27 (33.3%)
Delusions (suspiciousness/fear of harm)	32 (39.5%)
Hallucinatory behavior (self-muttering/laughing)	17 (20.9%)
Obsessive-compulsive symptoms (repetitive behavior)	17 (20.9%)
Verbal perseverance	27 (33.3%)
Addictive behaviour	18 (22.2%)
Suicidal tendencies	
Attempted	14 (17.2%)
Ideation only	4 (4.9%)
Family history of suicide	9 (11.1%)
*Cognitive symptoms*	37 (45.6%)
*Other Symptoms*	
Sleep disturbances	37 (45.6%)
Ongoing weight loss	40 (49.3%)
Reduced appetite	19 (23.4%)
Increased appetite	2 (2.5%)
Bladder incontinence	19 (23.4%)
Bowel incontinence	9 (11.1%)
Constipation	15 (18.5%)
Diabetes mellitus	9/74 (tested)
Hypothyroidism	12/68 (tested)

The mean UHDRS-TFC score was 7.5 ± 3.8. The score showed a significant negative correlation with duration of illness (correlation coefficient − 0.38, *p* < 0.0001), but not with age at disease onset (correlation coefficient − 0.073, *p* = 0.52) and CAG repeat length (correlation coefficient − 0.086, *p* = 0.45). Based on the TFC scores, the patients were categorized into 5 functional/disability stages as shown in Table 3.

**Table 3:** Staging based on TFC scores of study sample (*n* = 81)

Stage	TFC score	N (%)
I	11–13	18 (22.2%)
II	7–10	36 (44.4%)
III	3–6	16 (19.8%)
IV	1–2	6 (7.4%)
V	0	5 (6.2%)

Just over half the patients (46, 56.8%) were on regular follow-up (≥2 visit/year) since diagnosis, while 19 (23.5%) had irregular follow-up (≤1 visit/year), and the rest (*n* = 16, 19.8%) had been lost to follow-up. Among the 65 patients on follow-up, most (53; 65.4%) were being followed-up at NIMHANS, and the rest (12, 14.8%) at their local peripheral center. Most (64; 79%) were compliant with pharmacotherapy irrespective of follow-up visits, while 6 (7.4%) were poorly compliant and 11 (13.6%) had discontinued medications. Of the 70 patients taking medications, 63 (77.8%) were taking the prescribed medications, while the rest (7, 8.6%) were on alternative (Ayurvedic and Homeopathic) medications. About half the patients (40; 49.3%) were taking prescribed medicines on their own, while the rest (30, 37%) were being administered medications by care-givers.

Among the 17 patients who had died, the mean age at death was 53 ± 10.6 (range 40–67) years; and the mean duration of illness from onset of symptoms till death was 7 ± 2.9 (range 3–13) years. The mean CAG repeat length of these patients was 44 and it had a significant negative correlation with both age at onset (*p* < 0.001) and age at death (p < 0.001). The causes of death included suicide (3), falls resulting in subdural bleed (2), road traffic accident (1), multiple myeloma (1), and post-operative complications of meningioma (1). The remaining 9 patients developed complications due to the advanced disease such as cachexia, reduced food intake, aspiration pneumonia and systemic infections, which likely contributed to death.

## Discussion

Huntington’s disease has been diagnosed for more than half a century in India [12]. In this study, we outline the characteristics of HD patients diagnosed at our center, and describe the co-morbidity, mortality and access to care. The mean age at symptom onset (40.3 ± 10.4 years) and the mean CAG repeat length (44.2 ± 4.5 repeats) of our sample are comparable to the studies in Western populations [6, 17]. As expected, the age at symptom onset had significant inverse correlation with CAG repeat length.

Seventeen HD patients had died within 3 years of diagnosis (mean age at death of 53 years), none of them being institutional deaths. Though the mean duration of illness at death (7 years) is skewed by deaths due to trauma and cancer, it is considerably lower when compared to the mean survival of 24 years from diagnosis in a large European cohort (institutional deaths in 54% cases) [18]. Multiple factors could have contributed to this including delayed diagnoses, disease severity and inadequate care. Though the cause of death was unclear in many, most of the known causes (suicide, intracranial bleeds due to falls, aspiration pneumonia) are preventable. Quite often, families had been informed that this was an incurable condition. This pessimism, the lack of services, and the socio-economic factors (expenses involved in treatment of a person who was anyway ‘destined’ to die) perhaps contributed in no small part to the stark difference.

The estimated suicide rate in HD patients is 4–6 times higher than in the general population [19]. We too observed a high prevalence of suicidal tendencies (22%) in our sample, which is more than that was reported in other HD studies [20, 21] and that in the general Indian population (0.01%) [22]. Suicides resulted in 3 of the 17 deaths. Even greater concern is warranted as nine additional subjects also reported a family history of suicide in one or more members, involving both manifest HD and at-risk individuals. A disproportionately large number of patients with confirmed or suspected HD are thus ending their lives. The cause for suicidality in HD appears to be multifactorial [21, 23]. It may be part of the disease itself (depression or impulsivity), or may be secondary to disease-related disability, often influenced by socioeconomic and cultural factors [20]. This high rate of suicidal attempts and death may also reflect the degree of despondency and hopelessness that HD patients and their families experience.

The lifetime prevalence of psychiatric morbidity among HD patients varies between 33 and 76% as per various reports, with depression, apathy, irritability, aggressiveness, impulsiveness, obsessions, and compulsions being commonly reported [19, 24]. Nearly one-third patients in our sample reported non-motor symptoms at onset, and majority (91.4%) had psychiatric and behavioral problems, of varying severity, during the course of the disease. The most common problems were anger outbursts (65%), higher than previously reported (24), followed by low mood or depression (53%) and generalized anxiety (48%). A significant proportion (40%) also reported delusions, which is relatively high compared to previous studies in HD [24, 25]. These included delusions of infidelity, persecutory and somatic delusions. Apathy, not related to depression, was present in one-third patients, which is consistent with other studies [26]. Hallucinatory behavior (mostly visual) was noted in one-fifth of patients (relatively high) [25], while a similar proportion reported repetitive behavior like washing hands and ritualistic cleaning, suggestive of obsessive-compulsive disorder. The prevalence of addictive behavior (alcohol, tobacco and betel nut) in our sample (22%) is higher than that of general population [27]. Timely recognition of these problems facilitates appropriate treatment, with potential positive consequences on emotional, cognitive, and physical well-being.

Nearly half the patients also had cognitive dysfunction. It has been suggested that cognitive dysfunctions could account for as much as 70% of the variance in functional status [28]. Almost all subjects had chorea but majority of them were ambulant (91.4%), with no history of falls in more than half (56.8%) of them. There was high incidence of dysarthria (69%), and dysphagia (34.6%), which are important risk factors for death [29]. The overall assessment of the patients was often confounded by poor cooperation, bradykinesia, co-existing psychiatric conditions and cognitive dysfunction.

The low mean TFC score (7.5 ± 3.8) of the sample suggests significant functional disability. About two-third patients were classified as TFC stages I and II, a group potentially amenable to pharmacological therapies and multidisciplinary care. The other one-third patients had a TFC score below 7, indicating the need for palliative care. The TFC score had a significant inverse correlation with duration of illness, similar to other reports [30]. Care level scores were independent of domain specific disability and perhaps related to the resilience and stoicism of caretakers. Despite the physical and mental disability, majority of the patients were being looked after at home, without specialized nursing care. The long commute to access health care, poverty, lack of faith in treatment, estrangement from family, and disability discouraged a fifth of the patients from maintaining follow-up.

This small set of individuals, seen over 3 years, informs us of the complex needs of those with HD, and their families. Inadequate knowledge about HD amongst the health care professionals, lack of resources for HD families, and their unmet needs and the relation to low quality of life scores [31, 32] highlights the need to improve the quality of care, even in developed countries. Major limitation of our study is lack of quantitative analysis of the severity of various domains, their influence on each other and overall outcomes.

A conservative estimate of about 75,000 HD patients in India [10, 14] would indicate almost a million at risk. There is an urgent need to develop organized services for HD, and perhaps the entire range of adult-onset neurodegenerative conditions. Advances in genetic diagnostics will add to the number of ‘identifiable’ conditions in the near future, but specific treatments informed by this research are still a long way away. In the meantime, symptomatic treatments in line with international guidelines recommended by Bachoud-Lévi et al., [33], specialised counselling for different category of CAG repeats, especially in the wake of new advances in gene modifiers as recommended by Migliore et al., [34], adequate rehabilitative services that improve the quality of life, and overall outcomes, need to be developed and sustained.

## Conclusions

This study suggests that the symptomatic treatment is of great importance in HD, considering the morbidity and mortality due to potentially treatable psychiatric manifestations. Improving awareness regarding HD among the general public and the health-care professionals along with development of appropriate management strategies, and specialized structured multidisciplinary services, adapted to the needs of HD patients, can improve the follow-up rates. Though it is technically a ‘rare disorder’, public health provision has to keep in mind such diseases. The quality of services for those on the margins of the marginalized (as those with neuro-psychiatric illnesses often are) reflects the concern that society has for those with illness. Developing a national HD registry would enable us to determine the disease prevalence, facilitate systematic research, and develop better care for HD patients and their families; and thus serve as a model for other inherited disorders.

## Supplementary information

**Additional file 1: Supplementary file 1.** HD outcome study-Questionnaire. Description of data: This questionnaire contains parameters assessed for the consented participants which were used for clinical profiling along with clinical examination and medical record information.

## Data Availability

The datasets of the current study are available from the corresponding author on reasonable request.

## Abbreviations

HD: Huntington’s Disease
NIMHANS: National Institute of Mental Health and Neuro Sciences
TFC: Total Functional Capacity
UHDRS: Unified Huntington Disease Rating Scale
