# Supplementary material for: Psychiatric morbidity and poor follow-up underlie suboptimal functional and survival outcomes in Huntington’s disease
Source: BMC Neurol. 2020 Mar 12;20:87. doi: 10.1186/s12883-020-01671-x (PMC7068943; doi:10.1186/s12883-020-01671-x)
Supplement: Supplementary file 1 — Additional file 1: Supplementary file 1. HD outcome study-Questionnaire. Description of data: This questionnaire contains parameters assessed for the consented participants which were used for clinical profiling along with clinical examination and medical record information. [file 12883_2020_1671_MOESM1_ESM.docx]

HD outcome study-Questionnaire

Patient name: Age: Gender: education: occupation:

Informant: Relation of the informant: Duration of illness:

Last follow up date: Rate of follow up/year:

Reasons for losing follow up:

Medication intake: Allopathy/Alternative, self-administered/needs help of the care-taker

| Clinical parameter | Status/score |
| --- | --- |
| Index symptom/Symptom at onset/first abnormality noticed/:  Motor: abnormality body movements, walking difficulty psychiatric: personality changes/psychiatric diagnosis Cognitive: decreased skill sets, language impairment/memory issues | Motor/Psychiatric/cognitive |
| Current Total Functional Capacity:  OCCUPATION  0 = unable  1 = marginal work only  2 = reduced capacity for usual job  3 = normal  FINANCES  0 = unable  1 = major assistance  2 = slight assistance  3 = normal  DOMESTIC CHORES  0 = unable  1 = impaired  2 = normal  0 = total care  1 = gross tasks only  2 = minimal impairment  3 = normal  CARE LEVEL  0 = full time skilled nursing  1 = home or chronic care  2 = home | Total score= /13 |
| MOTOR SYMPTOMS | |
| Walking | Without support/with support/bed-ridden |
| Frequency of falls per week |  |
| Dysphagia: coughing while eating, swallowing problems | Yes/No |
| Dysarthria: change in quality of speech | Yes/No |
| Breathing difficulty: gasping for breath | Yes/No |
| Slowness in activities or responses | Yes/No |
| PSYCHIATRIC SYMPTOMS | |
| Generalized anxiety: worries, anticipation of the worst, fearful  anticipation. | Yes/No |
| Irritable Behavior: impatient, demanding, inflexible,  driven and impulsive, uncooperative | Yes/No |
| Aggressive behaviour/anger outbursts /abusive language | Yes/No |
| Depressive symptoms: low mood, crying spells, inability to enjoy usual interests | Yes/No |
| Suicidal symptoms | Nil/Ideation only/attempted |
| Delusional symptoms: Fear of harm, suspiciousness towards spouse or care taker, referential ideas | Yes/No |
| Hallucinatory behaviour: self-muttering, hearing of voices, seeing objects or persons | Yes/No |
| Addictive behaviour: substance usage (alcohol, tobacco, betel nut) | Yes/No |
| Apathy: unconcerned about family members, poor self care (not related to depressive symptoms mentioned above) | Yes/No |
| Verbal perseverance: Repeated utterance of same statement in quick succession | Yes/No |
| OCD spectrum symptoms: repeated washing of hands, objects. Preoccupation with one activity excessively | Yes/No |
| COGNITIVE SYMPTOMS | |
| Inability to concentrate/focus | Yes/No |
| Misplacing objects frequently | Yes/No |
| Forgetting names | Yes/No |
| Forgetting important events like birthdays, anniversaries, festivals | Yes/No |
| Counting/calculation difficulties | Yes/No |
| Decreased scholastic or occupational performance unrelated to motor symptoms | Yes/No |
| Way finding difficulty/Navigational difficulty | Yes/No |

SYSTEMIC SYMPTOMS

|  | Bladder incontinence | Yes/No |
| --- | --- | --- |
|  | Bowel incontinence | Yes/No |
|  | Constipation | Yes/No |
|  | Appetite | Normal/decreased/increased |
|  | Sleep disturbances | Yes/No |
|  | Weight loss | Ongoing/stabilized/nil |

Co-morbidities

|  | Diabetes Mellitus | Yes/No/borderline/not tested |
| --- | --- | --- |
|  | Hypothyroidism | Yes/No/not tested |
|  | Hypertension | Yes/No |
|  | others |  |

Family history of suicides in first degree relatives: yes/No, if yes, number of suicides ( )

Family history of cancers in first degree relatives: yes/No. if yes, type of cancer ( )

In case of death,

1. Age at death:
2. Duration of illness at the time of death
3. Cause of death
4. Whether hospitalized at the time of death or No
